# Does Executive Functioning Moderate the Association Between Psychopathic Traits and Antisocial Behavior in Youth?

**DOI:** 10.1007/s10802-024-01283-w

**Published:** 2025-01-25

**Authors:** Justin J. Joseph, Dan A. Waschbusch

**Affiliations:** 1 Department of Politics, Justice, Law, and Philosophy, University of North Alabama, Florence, AL USA; 2 Department of Psychiatry and Behavioral Health, Penn State College of Medicine, Hershey, PA USA

**Keywords:** Executive functioning, Psychopathy, Property crime, Violent crime

## Abstract

This study examined the interplay of psychopathic traits, executive functioning, and antisocial behavior among adjudicated youth, with a focus on the potential moderating role of executive function. The current study uses data from the Pathways to Desistance dataset was examined, utilizing the Psychopathy Checklist: Youth Version (PCL-YV) and the Stroop Color-Word Task to measure psychopathic traits and executive functioning, respectively. Violent and property offending frequencies were self-reported. Both psychopathic traits and lower executive functioning were initially associated with higher frequencies of both violent and property offending. Crucially, a significant interaction emerged: Youth exhibiting higher socially deviant/lifestyle psychopathic traits and weaker executive function were most likely to engage in property offenses. These findings offer insights into specific risk profiles for offending behaviors and underscore the importance of interventions promoting executive function, especially for youth with these characteristics. This study highlights the complex ways in which individual differences contribute to antisocial outcomes.

Antisocial behavior is a serious problem among adolescents. In the United States, approximately 1 in 14 adolescents will engage in serious forms of antisocial behavior, such as theft, violence, or property destruction (Puzzanchera, 2022). Antisocial behavior can have serious negative consequences for both the individual and society. Adolescents who engage in antisocial behavior are more likely to drop out of school and experience mental and physical health problems (Huesmann et al., 2009; Kim-Cohen et al., 2003). These youth are also more likely to be victimized, experience social isolation, and face economic hardship (Rivenbark et al., 2018). The Centers for Disease Control and Prevention (2023) estimates that youth violence costs $122 billion annually without including the cost of the criminal justice system.

Consistently, research has found that criminal behavior peaks during adolescence and significantly drops during adulthood; however, explanations for this age-crime curve phenomenon remain debated among scholars (Shulman et al., 2013). This study focuses on understanding the interplay between psychopathic traits, executive functioning, and antisocial behavior in youth, framed through Raine’s (2019) neuromoral theory, which provides a framework for understanding adolescent antisocial behavior from a developmental neuroscience perspective.

Neuromoral theory posits that specific neuroanatomical deficits, particularly in the prefrontal cortex, amygdala, and angular gyrus, impair proper moral development and contribute to antisocial behavior (Raine, 2019). These biological disruptions result in underdeveloped moral reasoning and conscience formation, leading to diminished capacity for emotional processing, moral decision-making, and prosocial behavior in affected individuals (Raine, 2019). While there is considerable heterogeneity in the presentation of these impairments, the most common manifestations are mild deficits that display more externalized symptoms, typically associated with adolescent-limited offenders or secondary psychopathy (Moffitt, 2018; Moffitt et al., 2013; Raine, 2019). These deficits particularly affect neural systems governing emotional regulation, behavioral inhibition, and integrating moral knowledge with emotional responses during critical developmental periods.

Adolescence represents a key period for examining these relationships, as it coincides with significant neurodevelopmental changes, particularly in the prefrontal cortex and its connection to limbic regions (Barker et al., 2007; Poon, 2018; Raine, 2019). During this period, executive functions are still maturing, potentially making adolescents more vulnerable to difficulties in impulse control, decision-making, and emotional regulation (Johnson et al., 2015; Poon, 2018; Raine, 2019). This developmental window is particularly relevant for understanding the relationship between executive functioning and psychopathic traits, as both constructs are influenced by the prefrontal cortex and its connection to emotional processing (Raine, 2019).

Psychopathic traits (PT) and executive functioning (EF) represent two key factors linked to antisocial behavior in adolescents, with distinct but potentially interactive influences. Psychopathic traits are characterized by a lack of empathy, remorse, and guilt, as well as impulsivity and aggression (Patrick, 2018). Executive functioning encompasses cognitive skills necessary for goal-directed behavior, such as planning, decision-making, and impulsive control. While numerous studies have separately examined PT and EF as predictors of antisocial behavior in youth, relatively little research has examined them together, particularly during the critical developmental period of adolescence (Bonham et al., 2022; Campbell et al., 2004; Forth & Book, 2010; Jansen & Franse, 2024). Understanding these relationships within the context of adolescent development is crucial, as this period represents both a time of risk and an opportunity for intervention.

Environmental factors also play a role in shaping antisocial behavior during adolescence. Gang involvement, adverse childhood experiences, economic hardship, maturing in economically disenfranchised neighborhoods, associating with delinquent peers, and a history of serious head injury have consistently been associated with more antisocial behavior amongst juvenile delinquents (Chui et al., 2023; McKinlay & Albicini, 2016; Sutton, 2017; Vecchio & Carson, 2023; Wood & Alleyne, 2010). Consistently, juvenile offenders report more vicarious and personal victimization experiences, serious head injury, and psychopathology in comparison to the general population (Livanou et al., 2019; McKinlay & Albicini, 2016; Turney, 2018). Moreover, cluster analysis studies have found that youth classified as secondary variants report more trauma and experiences of maturing in impoverished neighborhoods (Craig et al., 2021; Docherty et al., 2016; Kahn et al., 2013; Skeem et al., 2003), However, investigation into the interaction of psychopathic traits and executive functioning impact on adolescent antisocial behavior, while controlling for the impact these relevant variables is scant, warranting a more comprehensive investigation.

Studies suggest a complex interplay between psychopathy traits, antisocial behavior, and executive functioning. Children with both psychopathic traits and antisocial behavior exhibit a distinct profile in many domains, yet findings regarding their executive functioning are mixed. Specifically, some studies report better executive function performance for these youth compared to youth with antisocial behavior alone (Dotterer et al., 2021; Graziano et al., 2019; Waschbusch et al., 2022), while other studies report the opposite pattern (Hadjicharalambous & Fanti, 2018; Platje et al., 2018; Wall et al., 2016). Further, the potential moderating role of executive functioning on the relationship between psychopathic traits and antisocial behavior remains unclear. While some studies suggest better executive functioning may facilitate antisocial acts in youth with high psychopathic traits (Baskin-Sommers et al., 2015; Bonham et al., 2022; de Graaf et al., 2023; Muñoz et al., 2008), others indicate that worse executive functioning is associated with increased antisocial behavior in these individuals (Waller et al., 2017). Given these contradictions and the limited research, further investigation is needed to clarify the role of executive functioning.

This study, which aims to address these mixed findings, examines whether psychopathic traits, executive functioning, or their interaction are associated with antisocial behaviors in adjudicated youth while controlling for the influence of previously identified contributors to antisocial behavior. Based on the limited previous research, it was hypothesized that executive functioning would moderate the association between psychopathic traits and antisocial behavior such that youth with higher psychopathic traits and better executive functioning would exhibit more frequent antisocial behavior. This question was addressed using baseline data from the Pathways to Desistance study.

This study extends the work of Baskin-Sommers et al. (2015), which examined a similar question using the same dataset. It advances the research in three key ways. First, it specifically focuses on the frequency of violent and property offending rather than the variety of substance use and offending. This distinction is important, as different forms of delinquency may follow distinct etiological and developmental pathways (Loeber & Farrington, 2001; Loeber et al., 1998). Second, by incorporating a more comprehensive set of control variables, this study provides a more rigorous test of the core associations of interest. Third, this study includes both male and female participants and restricts the analysis to baseline data – the only time at which executive functioning was measured – thereby expanding beyond the previous study’s male-only sample and minimizing potential confounding effects related to developmental changes in executive functioning.

## Method

### Participants

This study used the publicly available data from the Pathways to Desistance dataset, a multi-site, longitudinal examination of serious adolescent offenders transitioning into adulthood; due to the public nature of the data IRB approval was not necessary (Mulvey, 2012). Data collection was sponsored by 10 agencies and intended to provide information on juvenile justice to policy-makers and justice officials. Originally designed to investigate social and psychological pathways to desistance (Mulvey, 2012), several previous studies have used this dataset to explore juvenile psychopathy (Boduszek et al., 2015; Dmitrieva et al., 2014). Participants consisted of 1,354 male and female adolescent juvenile delinquents, including 700 from Philadelphia, Pennsylvania, and 654 from Phoenix, Arizona. They were identified based on adjudication charge, age, and demographics to ensure a representative sample (Schubert et al., 2004). After appropriate consents were obtained, baseline evaluations occurred in the youth's home, an agreed-upon location, or the juvenile detention facility (see Schubert et al. (2004) for details of the methodology and data collection procedures). The current analysis included all participants who completed the Stroop Task at baseline (*n* = 1330). Table 1 summarizes the sample’s demographic characteristics and key variables.

**Table 1:** Sample descriptives

Variables	N	M	SD	%	Min	Max	MI
Age	1330	16.04	1.14		14	19	0
Victimization Witnessed	1330	3.77	1.20		0	7	0
Victimization Experienced	1330	1.57	1.46		0	6	0
Suppression of Aggression	1330	2.78	0.99		1	5	0
Consideration of Others	1330	3.49	0.89		1	5	0
Temperance	1330	2.88	0.86		1	5	0
Personal Rewards of Crime	1330	2.35	2.41		0	10	0
SES Status	1322	51.36	12.31		11	77	8
Neighborhood Conditions	1328	2.35	0.75		1	4	2
Delinquent Peer Influence	1318	1.77	0.86		0.52	5	12
Interpersonal/Affective	1283	5.06	3.51		0	20	47
Socially Deviant/Lifestyle	1283	8.34	3.87		0	21	47
Executive Functioning	1330	43.44	10.84		15	85	0
Violent Offending Frequency	1330	13.57	42.86		0	876	0
Income Offending Frequency	1330	104.7	301.48		0	3095	0
Race					0	1	0
White	270			20.30			
Hispanic	551			41.40			
Black	448			33.70			
Other	61			4.60			
Sex					1	2	0
Male	1148			86.30			
Female	182			13.70			
Head Injury					0	1	
Yes	401			30.10			
No	929			69.90			
Gang Membership					0	1	0
Yes	309			23.23			
No	1021			77.77			

*N* sample size, *M* mean, *SD* standard deviation, *%* percentage of sample, *Min* minimum value, *Max* maximum value, *MI* missing data count. All continuous variables are rounded to the nearest hundredth. SES Status is measured using parent education and occupation, with higher scores indicating lower socioeconomic status. Victimization variables are from the Exposure to Violence Inventory. Suppression of Aggression, Consideration of Others, and Temperance are subscales from the Weinberger Adjustment Inventory, with higher scores indicating more positive behavior. Interpersonal/Affective and Socially Deviant/Lifestyle scores are from the PCL:YV. Executive Functioning represents T-scores from the Stroop Color-Word Task, with higher scores indicating better performance. Offending Frequencies represent self-reported counts over the past six months

### Dependent Variables

#### Violent Offending Frequency

Violent offending frequency is represented by the sum of eleven aggressive offenses in the past six months, adapted from the Self-Reported Offending Inventory (Huizinga et al., 1991; Lee & Kim, 2022; Walters, 2015). These items include: 1) “destroyed/damaged property”, 2) “set fire”, 3) “forced someone to have sex”, 4) “murder”, 5) “shot someone”, 6) “shot at someone”, 7) “took by force with a weapon”, 8) took by force without a weapon”, 9) beat up someone resulting in serious injury”, 10) “participated in a fight”, and 11) “beat up someone as part of a gang”.

#### Property Offending Frequency.Offending Frequency

Property offending frequency is represented by the sum of ten income offenses in the past six months, also adapted from the Self-Reported Offending Inventory (Huizinga et al., 1991; Lee & Kim, 2022; Walters, 2015). The items include: 1) “broke in to steal”, 2) “shoplifted”, 3) “bought/received/sold stolen prop”, 4) “used check/credit card illegally”, 5) “stole care or motorcycle”, 6) “sold marijuana”, 7) “sold other drugs”, 8) “been paid by someone for sex”, 9) “took by force with a weapon”, 10) “took by force without a weapon”.

### Predictor Variables

#### Psychopathic Traits

The Psychopathy Checklist: Youth Version (PCL-VY: Forth et al., 2003) is a 20-item rating scale designed to assess the behavioral and personality components of psychopathy in adolescents. The PCL-YV is organized along two dimensions (Forth et al., 2003; Shepherd & Strand, 2016):

1) Interpersonal/Affective – reflecting callous-unemotional traits, lack of empathy, and superficial charm.

2) Socially Deviant/Lifestyle – reflecting impulsivity, rule-breaking, and a need for stimulation.

Items are scored using a 0 (not present at all) to 2 (present) metric. As suggested (Jones et al., 2006), items were summed to produce a factor score with a theoretical range from 0 to 40 (Mulvey et al., 2012). Numerous studies have supported this measure's psychometric properties (Forth & Book, 2007; Neumann et al., 2006). In the present sample, the Interpersonal/Affective subscale produced an alpha of 0.76 and intraclass correlation (ICC) of 0.79, and the Socially Deviant/Lifestyle subscale produced an alpha of 0.78 and an ICC of 0.93 (Mulvey, 2012).

#### Executive Functioning

Executive functioning was operationalized using the Stroop Color-Word Task, a well-established test that measures inhibitory control and cognitive flexibility (Golden, 1978; Swick & Jovanovic, 2002). This task is commonly used in studies of executive functioning (Nijdam et al., 2018), including studies with healthy and clinical participants (Baskin-Sommers et al., 2015; Cauffman et al., 2009). The task involves three conditions – word reading, color naming, and color-word interference – with participants completing as many items as possible within a 45-s time limit (Mulvey, 2012). Consistent with previous research (see Baskin-Sommers et al., 2015; Mulvey, 2012), standardized *T*-scores were computed using normative data, with higher scores reflecting better executive functioning and less interference effects (Golden, 1978).

### Control Variables

#### Exposure to Violence

Exposure to violence was assessed using the Exposure to Violence Inventory (ETV; Selner-O'Hagan et al., 1998). This scale measures both direct victimization and witnessing of violent events, providing separate subscales with well-supported psychometric properties (Mulvey et al., 2012). Exposure to violence during adolescence can significantly impact both executive functioning development and antisocial behavior trajectories (Op den Kelder et al., 2018).

#### Perceptions of Psychic Rewards of Crime

This concept was measured using the personal rewards subscale of the Indices of Personal and Social Costs and Rewards (Mulvey et al., 2012). This subscale assesses excitement or thrill associated with delinquency and has high internal consistency reliability (alpha = 0.88).

#### Neighborhood Conditions

Neighborhood conditions were measured using a self-report instrument adapted from Sampson and Raudenbush (1999) to assess physical and social disorder within the community. The scales demonstrate high internal consistency (total score alpha = 0.94; physical disorder alpha = 0.91; social disorder alpha = 0.87).

#### Peer Delinquency Influence

Peer Delinquency Influence was measured using the Peer Antisocial Influence subscale from the Rochester Youth Study (Thornberry et al., 1993). The subscale measures how strongly peers influence delinquent behavior, with high internal consistency reliability (alpha = 0.89).

#### Demographics

Demographic measures used as control variables included age, sex (1 = male, 2 = female), race (0 = White, 1 = Non-White), and socio-economic status (SES), operationalized using parent education and occupation (see Mulvey et al., 2013 for details), with higher scores indicating lower SES.

#### Gang Membership

Gang membership was measured by a single self-report item ("Have you ever been a member of a gang?") with a dichotomous response (0 = no, 1 = yes). This self-report is supported in previous research (Ang et al., 2015; Webb et al., 2006).

#### Head Injury

Head injury was assessed with descriptive items developed by neuropsychologists to establish the presence of brain injury. Participants were asked whether or not they experienced a head injury that resulted in the loss of consciousness or needed medical attention (Mulvey et al., 2012). The item was dichotomously coded (0 = No, 1 = Yes) and used to operationalize whether or not the participant had ever experienced a head injury. In the current sample, approximately 30% of participants report the previous head injury, which is within the estimated 12%—82% among adolescent and youth offender populations (McKinlay & Albicini, 2016).

#### Psychological Development

Psychological Development was measured using three subscales from the Weinberger Adjustment Inventory (WAI; Weinberger & Schwartz, 1990): temperance, suppression of aggression, and consideration of others. Higher scores on each subscale indicate more positive behavior (i.e., more impulse control, greater temperance, and greater consideration for others). The mean of the items that comprise the subscales were used as measures in the study. Higher scores on each of the subscales delineated below indicate more positive behavior (i.e., more impulse control, greater temperance, and greater consideration for others). For the current study, suppression of aggression, consideration of others, and temperance dimension were analyzed.

### Analytic Strategy

The relationship between psychopathic traits, executive functioning, and antisocial behavior was examined using robust negative binomial regressions as computed using the QuantPsyc and car packages R v 4.3. This approach was selected because dependent variables (income offending, violent offending) were frequency counts. Preliminary analyses suggested that multicollinearity was not a concern (variance inflation factors < 5). However, there was evidence of overdispersion of the data for both violent offending (z = 2.456, *p* < 0.001) and income offending (z = 5.765, *p* < 0.001), as well as violation of the multivariate normality assumption (Mardia Test: *β* = 192.593, *p* < 0.05). Therefore, a robust negative binomial regression was the most appropriate statistical model. Primary predictors of interest were psychopathic traits (PCL-YV facets), executive functioning (Stroop Task), and their interactions, along with the following control variables: age, race, sex, victimization witnessed, victimization experienced, gang membership, suppression of aggression, consideration of others, temperance, personal rewards of crime, SES status, neighborhood conditions, delinquent peer influence, head injury. Regressions were computed in two steps, with the main effects of psychopathy, executive functioning, and control variables entered in Step 1 and interactions entered in Step 2.

Missing data for most variables was addressed using the Expectation Maximization (EM) procedure (see Honaker et al., 2011) within the Amelia package in R. EM is a robust method for handling missing data within overdispersed count variables (Allison, 2002; Enders, 2003; McLachlan, 1997; Walters, 2022). Given the inherent characteristics of the Stroop Task and the minimal occurrence of missing data (*n* = 24; 1.7% of the original sample), listwise deletion was adopted for handling this missing data. Only baseline data was analyzed due to the availability of key variables (PCL-YV facets, Stroop Task). Additionally, it’s important to acknowledge the dynamic nature of human development and the potential for key measures to vary over time. Using baseline data minimizes confounding effects over the extended timescale of the longitudinal data.

## Results

### Violent Offending

The results of regression models examining the frequency of violent offending are summarized in Table 2.

**Table 2:** A negative binomial regression on violent offending frequency

Variables	Step 1		Step 2	
	IRR	S.E	IRR	S.E
Age	0.82***	0.03	0.82***	0.03
Hispanic	1.09	0.19	1.10	0.19
Black	1.00	0.15	1.00	0.15
Other	0.89	0.22	0.91	0.23
Sex	0.68**	0.09	0.69**	0.09
Victimization Witnessed	1.18***	0.04	1.17***	0.04
Victimization Experienced	1.07	0.05	1.08	0.05
Gang Membership	1.58*	0.28	1.56**	0.27
Suppression of Aggression	0.90	0.11	0.89	0.10
Consideration of Others	0.85*	0.06	0.84**	0.05
Temperance	0.86	0.13	0.88	0.12
Personal Rewards of Crime	1.09**	0.03	1.09***	0.03
SES Status	0.99	0.00	.99	0.00
Neighborhood Conditions	1.04	0.10	1.04	0.10
Delinquent Peer Influence	1.13	0.08	1.13	0.08
Interpersonal/Affective	1.05*	0.02	1.17	0.10
Socially Deviant/Lifestyle	1.08***	0.02	1.05	0.08
Head Injury	1.05	0.12	1.06	0.11
Executive Functioning	0.98**	0.01	0.99	0.01
Executive Functioning*Interpersonal/Affective			0.99	0.00
Executive Functioning*Socially Deviant/Lifestyle			1.00	0.00
AIC	8275.03		8274.28	
LoglikelihoodsHo	−4116.52		−4114.14	

*IRR* Incident Rate Ratio, *S.E* Standard Error, *AIC* Akaike Information Criterion. All integers are rounded to the nearest hundredth. Executive Functioning scores are standardized T-scores from the Stroop Task. PCL:YV Facets (Interpersonal/Affective and Socially Deviant/Lifestyle) are raw scores. Neighborhood Conditions scores are from the adapted Sampson and Raudenbush measure. Personal Rewards of Crime scores are from the Indices of Personal and Social Costs and Rewards. Delinquent Peer Influence is measured using the Peer Antisocial Influence subscale

a. Predictors entered on step 1: Race/Ethnicity (reference: White), Neighborhood Conditions, PCL:YV Facet 1 (Interpersonal/Affective), PCL:YV Facet 2 (Socially Deviant/Lifestyle), Stroop Task T-score, Personal Rewards of Crime, Delinquent Peer Influence, Violent Victimization, Violence Witnessed, SES Status, Age, Gang Membership (0 = no, 1 = yes), Suppression of Aggression (WAI), Consideration of Others (WAI), Temperance (WAI), Head Injury (0 = no, 1 = yes). Step 2 adds interaction terms between Executive Functioning and PCL:YV facets

b. Dependent Variable: Violent Offending Frequency

c. + *p* = .05, * *p* < .05, ** *p* < .01, *** *p* < .001

#### Psychopathic Traits

In Step 1, both the Interpersonal/Affective and Socially Defiant/Lifestyle facets of psychopathy were positively correlated with the frequency of violent offenses. Incident Rate Ratios (IRRs) of 1.05 (*p* < 0.05) were observed for each. A one-unit increase in the Interpersonal/Affective score was associated with a 5% increase in violent offenses and the same increase in the Socially Defiant/Lifestyle was associated with an 8% increase.

#### Executive Functioning

In Step 1, executive functioning was negatively correlated with the frequency of violent offenses, with an IRR of 0.98 (*p* < 0.05). A one-unit increase in the executive functioning score was associated with a 2% decrease in violent offending.

#### Interaction Effects

After including interaction terms in Step 2, the main effects of psychopathic traits and executive functioning were no longer statistically significant.

#### Control Variables. 

In both Step 1 and Step 2, age, exposure to violence, gang membership, and personal rewards of crime were consistently associated with higher violent offending

### Property Offending

**Fig. 1:**
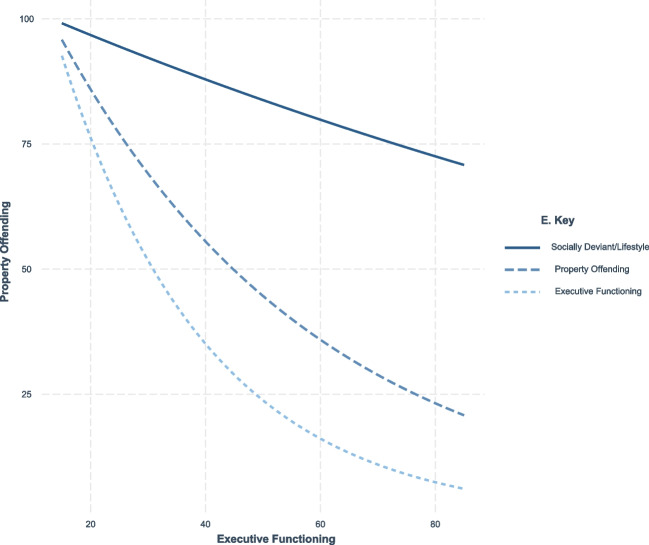
Interaction of psychopathy, executive functioning and property offending scores

Results of regression models examining the frequency of violent offending are summarized in Table 3 and Fig. 1.

**Table 3:** A negative binomial regression on income offending frequency

Variables	Step 1		Step 2	
	IRR	S.E	IRR	S.E
Age	1.39***	0.11	1.35***	0.11
Hispanic	0.77	0.21	0.73	0.20
Black	0.95	0.23	0.89	0.22
Other	0.69	0.32	0.69	0.34
Sex	0.81	0.19	0.81	0.20
Victimization Witnessed	1.14*	0.07	1.14*	0.07
Victimization Experienced	1.26**	0.09	1.23**	0.09
Gang Membership	1.03	0.21	1.08	0.22
Suppression of Aggression	0.94	0.14	0.91	0.14
Consideration of Others	.71***	0.07	.72***	0.07
Temperance	1.17	0.26	1.22	0.27
Personal Rewards of Crime	0.99	0.04	1.00	0.04
SES Status	0.99	0.01	0.99	0.01
Neighborhood Conditions	1.42**	0.15	1.42**	0.16
Delinquent Peer Influence	1.18*	0.10	1.15	0.10
Interpersonal/Affective	1.06*	0.03	0.96	0.10
Socially Deviant/Lifestyle	1.14***	0.03	0.94	0.08
Head Injury	1.35	0.26	1.42	0.28
Executive Functioning	0.98**	0.01	0.93	0.02
Interpersonal/Affective*Executive Functioning			1.00	0.00
Socially Deviant/Lifestyle*Executive Functioning			1.00*	0.00
AIC	10,911.65		10,904.25	
LoglikelihoodsHo	−5434.83		−5429.13	

*IRR* Incident Rate Ratio, *S.E* Standard Error, *AIC* Akaike Information Criterion. All integers are rounded to the nearest hundredth. Income offending frequency represents the sum of self-reported property and financial crimes over six months. Executive Functioning scores are standardized T-scores from the Stroop Task. PCL:YV Facets (Interpersonal/Affective and Socially Deviant/Lifestyle) are raw scores. All WAI subscales (Suppression of Aggression, Consideration of Others, Temperance) use mean scores with higher values indicating better adjustment

^a^Predictors entered in Step 1: Race/Ethnicity (reference: White), Neighborhood Conditions, PCL:YV Facet 1 (Interpersonal/Affective), PCL:YV Facet 2 (Socially Deviant/Lifestyle), Stroop Task T-score, Personal Rewards of Crime, Delinquent Peer Influence, Violent Victimization, Violence Witnessed, SES Status, Age, Gang Membership (0 = no, 1 = yes), Suppression of Aggression (WAI), Consideration of Others (WAI), Temperance (WAI), Head Injury (0 = no, 1 = yes). Step 2 adds interaction terms between Executive Functioning and PCL:YV facets

^b^Dependent Variable: Income Offending Frequency

^c^ + *p* = .05, * *p* < .05, ** *p* < .01, *** *p* < .001

#### Psychopathic Traits

In Step 1, both facets of psychopathy were positively associated with property offending frequency, with IRRs of 1.06 (*p* < 0.05) for Interpersonal/Affective and 1.14 (*p* < 0.05) for Socially Deviant/Lifestyle. A one-unit increase in the Interpersonal/Affective score was associated with a 6% increase in property offenses and the same increase in the Socially Defiant/Lifestyle was associated with a 14% increase.

#### Executive Functioning. 

In Step 1, executive functioning was negatively correlated with the frequency of violent offenses, with an IRR of 0.98 (*p* < 0.05). A one-unit increase in the executive functioning score was associated with a 2% decrease in violent offending.

#### Interaction Effects

In Step 2, there was a significant interaction between the Socially Deviant/Lifestyle factor and executive functioning on property offending (see Fig. 1). Specifically, youth scoring higher on this psychopathy dimension and lower on executive functioning demonstrated a 0.4% increase in property offending.

#### Control Variables

In both Step 1 and Step 2, age, exposure to violence, consideration of others, and neighborhood conditions were significantly associated with the number of property offenses perpetrated in the past six months.

## Discussion

This study investigated the relationships between executive functioning, psychopathic traits, and antisocial behavior, specifically focusing on the potential moderating role of executive functioning. By framing our investigation within Raine’s neuralmoral theory and controlling for relevant factors such as gang membership, peer delinquency, neighborhood conditions, and age, this study adds new insights to our understanding of these complex interactions. Initial findings indicated that both higher psychopathy traits and lower executive functioning were significantly associated with higher frequencies of violent and property offending. However, within the full models, it was only the interaction between the Socially Deviant/Lifestyle dimension of psychopathy and executive functioning that significantly predicted property offending. These findings align with existing literature linking psychopathy and executive dysfunction with antisocial behavior (Asscher et al., 2011; DeLisi, 2016; Fanti et al., 2016; Gil-Fenoy et al., 2018; Hampton et al., 2014; Lee & Kim, 2022; Ogilvie et al., 2011; Salekin & Andershed, 2022; Walters, 2014).

### Psychopathic Traits, Executive Functioning, and Antisocial Behavior in Development

The interaction effect suggests that youth higher in socially deviant traits and exhibiting poorer executive functioning are most prone to property offending. This finding can be understood through Raine’s (2019) neuromoral theory, which suggests that neurobiological deficits, particularly in the prefrontal cortex and its connections to emotional processing regions, can create a cascade of effects during adolescent development. These deficits may be particularly impactful during adolescence when executive functions are still maturing and neural plasticity is high (Johnson et al., 2015). Youth with mild neurological impairments often display more externalized symptoms (e.g., poor emotional regulation, impulsivity, reactive aggression), which overlap with characteristics observed in juveniles identified as secondary psychopaths (Docherty et al., 2016; Kimonis et al., 2011, 2012).

Previous work has identified that secondary psychopathy in both juvenile and adult samples is characterized by higher scores on the socially deviant dimension, coupled with histories of adverse childhood experiences and development in socially disorganized neighborhoods (Docherty et al., 2016; Kahn et al., 2013; Skeem et al., 2003). These results are consistent with findings from studies of adults that executive functioning deficits are uniquely and specifically associated with the socially deviant/lifestyle dimension of psychopathy (Poythress & Hall, 2011; Ross et al., 2007).

The distinction between primary and secondary psychopathy may be particularly relevant during adolescence, as it may reflect different developmental pathways to antisocial behavior ( equifinality). Primary psychopathy is described as primarily congenitally or biologically driven, whereas secondary psychopathy appears more environmentally driven (Porter, 1996). Research suggests that individuals with secondary psychopathy are characterized by mild neurological impairments, including in the polar, medial, and ventral prefrontal cortices, as well as elevated levels of impulsivity, anxiety, and hypersensitivity to stress (Moffitt, 2018; Raine, 2019). These characteristics may make them particularly vulnerable during adolescence, a period of both heightened stress sensitivity and ongoing prefrontal development, putting them at risk for opportunistic property crimes.

Our findings also align with research on adolescent-limited conduct disorder, which is highly associated with property-related offenses (Loeber & Stouthamer-Loeber, 1998). The median age of onset for property-related offenses is approximately 12 years (Loeber et al., 1993), coinciding with rapid changes in executive functioning (Barker et al., 2007). These results are consistent with longitudinal studies demonstrating that poor executive functioning in childhood predicts multiple adverse adolescent outcomes, including an increased likelihood of drug use and adverse life outcomes (Doan et al., 2019; Handley et al., 2017; Moffit et al., 2013).

### Clinical Implications

These findings highlight the potential importance of targeting executive functioning in interventions for youth with psychopathic traits who have perpetrated serious offenses, particularly during this critical developmental window. Cognitive remediation, designed to improve inhibitory control, could be especially beneficial for youth with psychopathic traits who have perpetrated serious offenses resembling the secondary psychopathy profile. Some research supports its potential for adults with psychopathic traits (Baskin-Sommer et al., 2015), suggesting it may be particularly effective during adolescence when neural plasticity is high. Moreover, early interventions focusing on parent training, social skills development, and family therapy might promote healthy prefrontal cortex development, ultimately reducing antisocial behavior tendencies (Junewicz & Billick, 2020). These types of personalized interventions may be crucial for effectively treating antisocial behavior in youth that have perpetrated serious offenses (Ng & Weisz, 2016).

### Limitations and Future Directions

Several limitations should be considered when interpreting these findings. First, while our focus on serious offenders in the Pathways sample provides important insights into this high-risk population, it limits generalizability to youth involved in less severe offenses. Therefore, it is important to remember that these findings are specifically applicable to serious offenders, who represent a small percentage of youth offerncers overall.

Second, our study relies on a single measure to operationalize executive functioning, which limits the ecological validity of the findings and our ability to effectively measure the complex and multifaceted nature of EF. The use of a single test cannot definitively indicate EF deficits; verification using multiple EF measures and neuroimaging would provide a more comprehensive picture of the role of EF in psychopathy and functioning. Future research should employ multiple measures of EF to capture its various components and their potentially distinct relationships with psychopathic traits and antisocial behavior.

Third, the lack of repeated measures of key variables (PCL-R and Stroop Task) across time points prevents proper longitudinal analysis. While it might be tempting to examine relationships between these variables at later time points, this would be inappropriate given that executive functioning continues developing until approximately age 30, while psychopathic traits show relative stability for some youth (Frick, 2018; Ferguson et al., 2021). Future research should employ longitudinal designs with repeated measures of both executive functioning and psychopathic traits to better understand their developmental trajectories and interactions.

Despite the limitations, the study has several strengths. It employs well-validated measures and rigorous statistical analyses, adding insight into the interplay of executive functioning, psychopathy, and antisocial behavior. Notably, the findings build on previous research by using a large sample size and incorporating control variables that influence the outcomes and may be confounded in previous studies. The current study also contributes to understanding the age crime curve from a biosocial perspective.

Future research should continue this line of investigation using longitudinal designs to replicate and extend these findings. Investigating how executive functioning moderates the relationship between psychopathy and antisocial behavior across development offers a particularly promising avenue of research. Additionally, studies should carefully consider sex differences across offending patterns and analyze sex for a more comprehensive understanding. Finally, this study underscores the need for innovative, evidence-based interventions targeting the underlying processes associated with youth psychopathy. Given the mixed evidence on current treatment approaches (da Silva et al., 2024), exploring interventions focused explicitly on enhancing executive functioning in youth with socially deviant aspects of psychopathy during this critical developmental period could prove particularly beneficial.
